# Tumor BRCA testing in ovarian cancer and EQA scheme: our experience of a critical evaluation

**DOI:** 10.1007/s11033-021-06812-0

**Published:** 2021-10-13

**Authors:** Elisa De Paolis, Paola Concolino, Maria Elisabetta Onori, Concetta Santonocito, Claudia Marchetti, Anna Fagotti, Giovanni Scambia, Andrea Urbani, Angelo Minucci

**Affiliations:** 1 Molecular and Genomic Diagnostics Unit (MGDUnit), Fondazione Policlinico Universitario A. Gemelli IRCCS, Rome, Italy; 2 Division of Oncological Gynecology, Department of Women’s and Children’s Health, Fondazione Policlinico Universitario A. Gemelli IRCCS, Rome, Italy; 3 Università Cattolica del Sacro Cuore, Rome, Italy; 4 Molecular and Genomic Diagnostics Unit (MGDUnit), Fondazione Policlinico Universitario Agostino Gemelli IRCCS, Università Cattolica del Sacro Cuore, Rome, Italy

**Keywords:** Next generation sequencing, Tumor *BRCA* testing, Ovarian cancer, EMQN, EQA, Allelic dropout

## Abstract

Next generation sequencing (NGS) is a widespread molecular biology method integrated into clinical practice to detect genetic variants, for diagnostic and prognostic purposes. The scheduled external quality assessments (EQA) is integral part of clinical molecular laboratory quality assurance. The EQA provides an efficient system to compare analytic test performances among different laboratories, which is essential to evaluate consistency of molecular test. EQA failures demands targeted corrective action plans. In this context, the complexity of the NGS techniques requires careful and continuous quality control procedures. We report a tumor *BRCA1/2 (tBRCA)* testing benchmark discrepancy provided by the European Molecular Genetics Quality Network in our laboratory during a round of EQA for somatic mutation testing of *BRCA* genes in relation to ovarian cancer. The critical analysis emerging from the *tBRCA* EQA is presented. We underline that harmonization processes are still required for the EQA in the molecular biology field, especially if applied to the evaluation of methods characterized by high complexity.

## Introduction

Next generation sequencing (NGS) are beginning to replace traditional molecular techniques [1]. Giving the implementation of NGS in clinical settings, best practice guidelines are now available to introduce quality management processes. Quality management includes policies established with the aim to provide accurate laboratory test, as internal quality control and external quality assessment (EQA) programs. In particular, EQA involves the examination of the laboratory procedures by a third-party accreditation process, and are available for the majority of well-established technologies [2]. It is an inter-laboratory comparison that may extend throughout all phases of a testing cycle including interpretation of results [3]. EQA provides the examination of different types of external samples depending on the scheme version. For the germline scheme, laboratories generally receive genomic DNA extracted from cell lines. In somatic schemes, genetically engineered samples are generally provided. These artificial controls can be obtained by homogenously mixing mutant versus wild-type (WT) cell lines at defined allelic ratios, which closely mimics the formalin-fixed paraffin-embedded (FFPE) tissue block [4].

### Tumor BRCA testing in ovarian cancer and EQA scheme: experience in our Molecular and Genomic Diagnostics Unit

In order to evaluate tumor *BRCA1/2 (tBRCA)* testing performed in our Molecular and Genomic Diagnostics Unit (MGDUnit) as a predictive biomarker of response to platinum-based-chemotherapy and PARP-inhibitors in ovarian cancer patients [5], we recently joined the disease-based Somatic Ovarian 2020 scheme of the European Molecular Genetics Quality Network (EMQN) [6]. The EMQN offers disease-specific and method-based EQA schemes when participating once a year to three different samples per EQA exercise. Participants are assessed on their ability to correctly genotype, interpret, and report the molecular results using their usual laboratory report format [2].

Our MGDUnit routinely assesses the *BRCA* molecular status using the amplicon-based library preparation kit Devyser *BRCA* (Devyser, Stockholm, Sweden) on Illumina MiSeq NGS platform [7, 8].

After uploading the EMQN scheme results, a discrepancy emerged between our *BRCA* genotyping and those expected in one of the samples provided by the scheme.

The validated sample genotype declared the presence of the *BRCA1* NG_005905.2 (NM_007294.4):*c.68_69del* (rs80357914, p.(Glu23ValfsTer17)), variant with an expected variant allele frequency of 20 %. Our NGS analysis failed to identify the presence of this variant in the sample.

Despite this evidence, we received an EMQN communication that explains the observed controversial result. The artificial FFPE tumor sample provided contained a cell line engineered to generate the *BRCA1 c.68_69del* variant. The engineering process used to generate the variant (undertaken by Horizon Discovery, Cambridge, UK), left a small piece of artificial DNA (the “engineering scar”) of approximately 90 bp in the intron upstream of the variant. The insertion site was positioned at the *BRCA1 c.80+48_ c.80+49* site and caused the mis-priming of a PCR primer used by the Deyvser *BRCA* kit (Devyser AB, Stockholm, Sweden). Consequently, this engineering “scar” meant that this kit was unable to detect the *BRCA1 c.68_69del* variant for any laboratory using. This could potentially lead a laboratory to fail the EQA scheme; however, this did not occur because EMQN had detected the error during their assessment process and no laboratory using this kit was penalized. The technology used to make this artificial material has subsequently been superseded by the use of CRISPR system, which does not leave an engineering “scar”.

### Question: Why did the Devyser kit not identify the c.68_69del variant in the EQA sample?

Taking into account that poor performance in the EQA represents a relevant alert, we decided to explore the discrepancy experienced in the EMQN exercise. In particular, we reported the experimental workflow adopted to evaluate the EMQN *BRCA1* genotype via alternative methods considering the technical information about the artificially development of the external sample. We undertook this effort to exclude that the failure in the identification of the EMQN *BRCA1* variant, attributable to the analytical incompatibility of the EQA scheme and the Devyser kit, cannot occur in human tissue samples in an appropriate clinical setting.

**Fig. 1:**
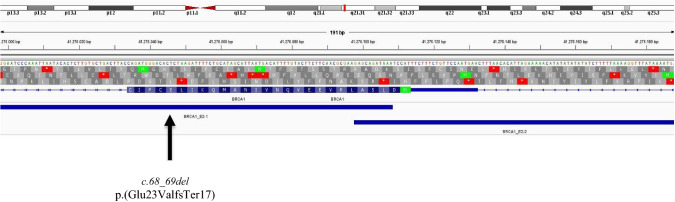
Integrative genomics viewer (IGV) visualizations of the *BRCA1* sequence surrounding the region of interest (Human hg19) and containing the two partially overlapping Devyser amplicons BRCA1_E2-1 and BRCA1_E2-2

**Fig. 2:**
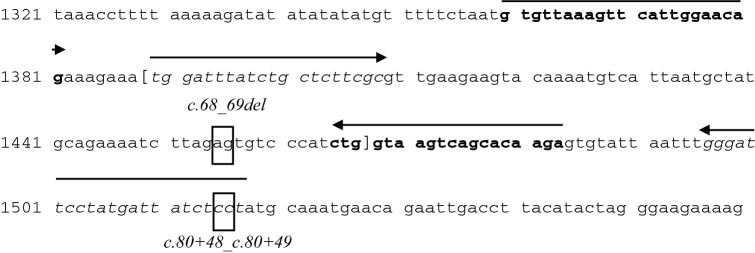
The *BRCA1* sequence surrounding the region of interest (LRG_292t1) is showed, with the exon 2 boundaries reported in bracket. The declared *BRCA1* variant *c.68_69del* and the insertion site *c.80+48_c.80+49* are also depicted (boxes). The primer pair designed for the first Sanger se-quencing reaction are reported in italic as: forward: 5′-TGGATTTATCTG CTCTTCGC-3′; reverse: 5′-AGGAGATAATCATAGGAATCCC-3′. The primer pair used in the second Sanger sequencing reaction are reported in bold as: forward: 5′-GTGTTAAAGTTCATTGGAACAG-3′; reverse: 5′-TCT TGT GCT GAC TTA CCAG-3′. The latter allow the correct identification of the BRCA1 variant. The primers were designed by Primers 3 software (http://primer3.ut.ee/)

The *c.68_69del* variant falls into the exon 2 of the *BRCA1* gene. The Devyser *BRCA* kit amplifies this region via two different amplicons, which are only partially overlapping. The *BRCA1_E2-1* amplicon has the sense primer that matches the region including the insertion site *c.80+48_ c.80+49*, precluding its effective amplification (Fig. 1). Because the NGS results were obtained only by the WT allele, as a consequence of the allelic dropout (ADO), we missed the sequencing information regarding the *c.68_69del* allele. We decided to better understand the type of molecular lesion affecting the specific *BRCA1* region. We firstly designed *ad hoc* primer pair including the *c.80+48_c.80+49* site (Fig. 2). Sanger sequencing of this PCR product highlighted again a WT sequence demonstrating that the engineering extends beyond the insertion site (at least up to the antisense primer). Therefore, this molecular alteration cannot occur in extracted human genomic DNA samples. However, since some *BRCA1* polymorphisms are reported in the *c.80+48_c.80+49* position (e.g. *c.80+48 C>T*, rs200513210, and *c.80+49 C>G* variants), the possibility of a failure in the amplification of this region using the Devyser *BRCA* kit remains open. Certainly, the minor allele frequency of these polymorphisms seems to be very low and they are never reported in linkage with the *c.68_69del* variant.

## Results

### Identification of the c.68_69del variant by alternative PCR/NGS approach

**Fig. 3:**
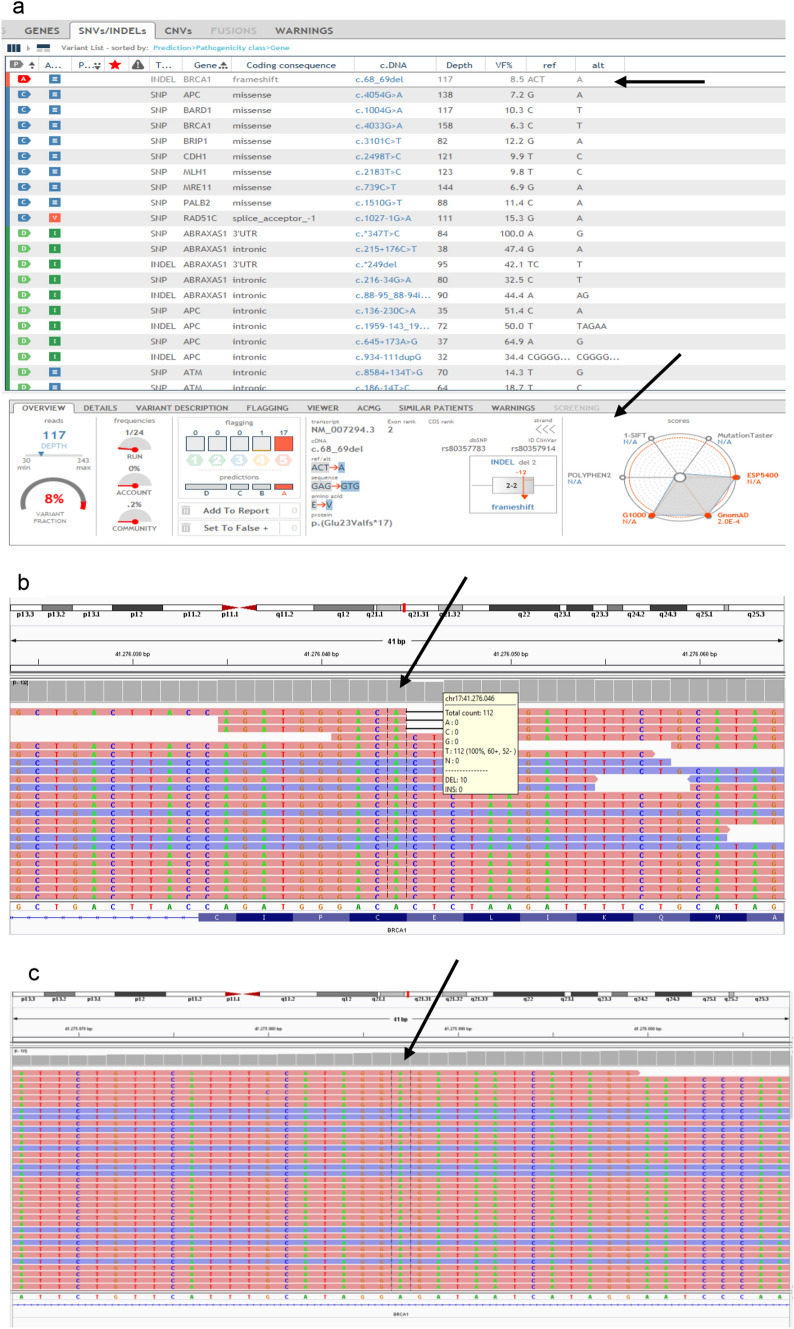
**a** The analysis performed by using the Sophia Genetics DDM software v3.4.0–4.6.2 (Sophia Genetics, Saint-Sulpice, Switzerland) for the *BRCA1 c.68_69del* (rs80357914) variant is reported, together with the associated variant information (black arrows). Integrative Genomics Viewer (IGV) visualizations of the BAM files obtained by using the Hereditary Cancer Solution CE-IVD kit (SOPHiA Genetics, Saint-Sulpice, Switzerland) are shown to highlight; **b** the coverage result and the reads data associated to the *BRCA1 c.68_69del* variant (black arrow); **c** the wild-type sequence obtained for the *BRCA1* region that includes the insertion site *c.80+48_c.80+49* (black arrow). This evidence suggests that both amplification and sequencing involved only the wild-type allele

Since the Devyser kit was unable to identify the *c.68_69del* variant, we investigated and confirmed the presence of the declared *BRCA1* variant using an alternative PCR/NGS molecular approach. In particular, we adopted the Hereditary Cancer Solution CE-IVD kit (SOPHiA Genetics, Saint-Sulpice, Switzerland), a NGS capture-based target enrichment of 26 cancer related genes (*ATM, APC, BARD1, BRCA1, BRCA2, BRIP1, CDH1, CHEK2, EPCAM, FAM175A, MLH1, MRE11A, MSH2, MSH6, MUTYH, NBN, PALB2, PIK3CA, PMS2, PMS2C, PTEN, RAD50, RAD51C, RAD51D, STK11, TP53, XRCC2*). As shown in Fig. 3a, this kit correctly detected the *c.68_69del* variant (showing a variant allele frequency of about 8 %). In addition, NGS data provides also information regarding part of the *BRCA1* intron 2, including the *c.80+48_c.80+49* site. Analyzing NGS results for this region (Fig. 3), a WT sequence was observed highlighting that the WT allele had been preferentially sequenced and confirming that the vector insertion region is wide extending beyond the *c.80+48_c.80+49* site, as already assumed after performing Sanger sequencing.

### Confirmation of the c.68_69del variant via by an orthogonal method: a targeted Sanger sequencing

**Fig. 4:**
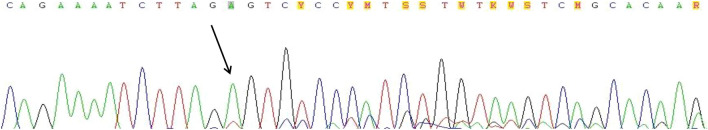
Sanger sequencing result of the *BRCA1* exon 2 region surrounding the EMQN declared variant (LRG_292t1). The presence of the *BRCA1 c.68_69del* variant at low VAF was depicted with a clear shift of the nucleotide sequence from the position *c.68 A* (black arrow). Sanger sequencing was performed with the Applied Biosystems 3500 Genetic Analyzer (Life Technologies, Carlsbad, CA, USA)

In order to confirm the presence of the *BRCA1 c.68_69del* variant via Sanger sequencing, we designed an *ad hoc* PCR primer pair that ensured the amplification of the mutated *c.68_69del* allele. The antisense primer was designed to pair in the exon/intron junction of the *BRCA1* exon 2, before the *c.80+48_c.80+49* site generating a small fragment, compatible with DNA quality from FFPE material (Fig. 2). As shown in Fig. 4, this strategy allowed us to correctly identify the *c.68_69del* variant in the EQA sample.

### Concluding remarks

During an EQA program from EMQN for *tBRCA* testing, our MGDUnit failed to identify the somatic *BRCA1 c.68_69del* variant. All laboratories that used the Deyvser *BRCA* kit obtained the same result. The EMQN did not assign the category of poor performance to any laboratory that failed to detect the *c.68_69del* variant using this kit. They acknowledged that the material used contained an artificial engineering scar that caused mis-priming of a PCR primer. We decided to explore this result using alternative methods encompassing different PCR/NGS approaches in addition to the traditional Sanger sequencing technique. Firstly, an attempt using Sanger sequencing was made to identify the vector insertion region without success. Secondly, a different PCR/NGS method was used allowing to correctly identify the EMQN variant declared. Third, we confirmed the presence of the EMQN *c.68_69del* variant using a targeted Sanger sequencing designing *ad hoc* an PCR/sequencing primer pair.

Sequencing of *BRCA* genes is the prominent approach for routine screening of genomic variations in ovary, breast, pancreas and prostate cancer patients [9, 10]. Participation to EQA allows monitoring of laboratory performance in an inter-laboratory level. In addition, EQA participation is an integral part of the quality framework of diagnostic laboratories, required by the International Organization for Standardization (ISO 15,189) [11].

A crucial aspect of EQA is the nature of the external source of quality control material. The ideal EQA sample should be obtained from clinical specimens, most closely representing real testing in a clinical laboratory [2]. However, the use of FFPE clinical tissue samples for large-scale formal EQA studies is nearly impossible due to the limited amount of tissue samples available from a given patient. In addition, the fixation process usually yields degraded DNA and sequence artifacts are frequently detected. Consequently, appropriate quantities of high-quality and stable FFPE clinical EQA samples are difficult to obtain [2]. In light of these critical issues, artificial samples, engineered to contain variants of interest, are often used in EQA schemes [4]. Here, we underlined that the DNA sequences flanking the mutations of interest in the artificial fragment must be identical to the *in vivo* genomic DNA sequences. In fact, this feature should allow to confirm the NGS results using a Sanger sequencing approach. In this study, only *ad hoc* Sanger sequencing allowed confirming the EMQN NGS results.

Moreover, as here described, some anecdotal evidences reported in literature indicate that NGS could not always optimally perform with DNA isolated using some external source [2] and errors, commonly associated to the assay design, could occur. ADO is a common phenomenon that reduces the efficiency of PCR-based targeted sequencing. It was first described as a “partial amplification failure” causing a potential source of misdiagnosis for both dominant and recessive diseases [12]. The ADO phenomenon involves selective allele amplification during the PCR process. The presence of single nucleotide variants in primer binding sites may lead to the complete or partial lack of amplification of the single allele, while the second may “drop” out during the PCR. Generally, the amplicon-based NGS strategy consists in multiplex PCR producing overlapping amplicons and providing a full and uniform coverage of all exons and exon/intron junctions of genes. This method assures a higher coverage of insertions/deletions and also an accurate analysis of copy number variants. However, the ADO remains one of the most relevant technical limitation of different PCR-based methods, as depicted elsewhere [13]. Moreover, several cases of unrecognized polymorphic variants located in the binding sites of the amplification primer that preclude the detection of specific mutations, are reported in literature. For example, one EQA scheme for Cystic Fibrosis evidenced that a laboratory-developed test could not accurately detect the *c.621+1G>T* mutation declared in the quality assessment [14]. Similar evidence was reported for *BRCA* testing [15] and for hereditary hemochromatosis [16]. In all these cases, the involved laboratories used the observed discrepancies as an opportunity to review their analytical process. The experienced ADO caused by the mutagenesis method using the Devyser *BRCA* kit reinforce the need of a careful optimization of the NGS workflow. In our Devyser *BRCA* NGS diagnostic workflow, the monitoring of ADO events is obtained via the identification of a strong and deep deletion signal obtained in the NGS copy number variant evaluation involving an amplicon among all the overlapping amplicons covering one exon. In this type of suggestive situation, we perform an alternative NGS test using a capture-based method, as described for the purpose of this paper.

Considering that the library preparation kit used in our molecular workflow is an amplicon-based type, at first we suspected the occurring of an ADO event. However, in the light of what has been reported, the ADO turns out to be exclusively dependent on the artificial nature of the external EMQN sample. Our efforts to understand the unexpected EQA scheme result, underlined the educational aspect of the EQA process, which generally helps laboratories to detect errors in their protocols and adopt corrective strategies improving the molecular assay outcome.

### Summary

In this paper, we focused on an EMQN scheme used for the EQA of somatic variant detection in *BRCA* genes. In this context, our MGDUnit experienced a failure in EQA performance. However, a careful and critical evaluation of this error help us to better understand if corrective actions, as redesigning tests or adjustment of methods, are needed.

We underlined that the adoption of complex testing methods, as NGS, should require implementation or optimization of the actual EQA design. Ideally, major attention should be paid to the plethora of amplification and NGS approaches available, ensuring that the success of an EQA is independent from the type of NGS or library preparation approach used.

New EQA approaches could be useful to ensure the continued quality in case of advanced and complex technologies. For example, a new prospective emerges from EQA schemes that use FFPE samples derived from untransformed cells modified using the CRISPR/Cas9 system without dramatically altering the original DNA sequence [2].

In conclusion, an appropriate EQA material could be useful to assure high-quality NGS testing in clinical laboratory settings. With this regard, each laboratory should take part to EQA schemes in order to critically analyze and improve the quality of own work. We would like to point out that a critical approach is always recommended to test the laboratory skills and abilities.

## Abbreviations

NGS: Next generation sequencing
EQA: External quality assessment
WT: Wild-type
FFPE: Formalin-fixed paraffin-embedded
tBRCA: Tumor BRCA1/2
MGDUnit: Molecular and Genomic Diagnostics Unit
EMQN: European Molecular Genetics Quality Network
ADO: Allelic dropout
ISO: International Organization for Standardization
